# Melatonin Regulates Root Architecture by Modulating Auxin Response in Rice

**DOI:** 10.3389/fpls.2017.00134

**Published:** 2017-02-07

**Authors:** Chengzhen Liang, Aifu Li, Hua Yu, Wenzhen Li, Chengzhi Liang, Sandui Guo, Rui Zhang, Chengcai Chu

**Affiliations:** ^1^Biotechnology Research Institute, Chinese Academy of Agricultural SciencesBeijing, China; ^2^National Center for Plant Gene Research (Beijing), State Key Laboratory of Plant Genomics, Institute of Genetics and Developmental Biology, Chinese Academy of SciencesBeijing, China

**Keywords:** root growth and development, root architecture, melatonin, auxin, transcriptome, rice

## Abstract

It has been suggested that melatonin acts as an important regulator in controlling root growth and development, but the underlying molecular mechanism driving this relationship remains undetermined. In this study, we demonstrated that melatonin acts as a potent molecule to govern root architecture in rice. Treatments with melatonin significantly inhibited embryonic root growth, and promoted lateral root formation and development. Genome-wide expression profiling by RNA-sequencing revealed auxin-related genes were significantly activated under melatonin treatment. Moreover, several transcription factors and candidate *cis*-regulatory elements involved in root growth and developments, as well as auxin-related processes, were over-represented in both co-up and -down differentially expressed genes, suggesting that melatonin-mediated root growth occurs in an auxin signal pathway-dependent manner. Further, gravitropic response analysis determined that melatonin affects auxin-regulated processes in rice root. These data show that melatonin shapes root architecture by directly or indirectly activating the auxin signaling pathway.

## Introduction

Melatonin (*N*-acety-5-methoxytryptamine) is highly conserved, biologically active molecule, presents in all eukaryotic organisms including fungi, mosses, plants, and animals (Tan et al., 1993; Reiter et al., 2014; Schippers and Nichols, 2014). It is best known as a neurohormone that controls circadian physiology and seasonal behavior in animals (Dollins et al., 1994; Karasek, 2004; Tosches et al., 2014). Recently, numerous studies revealed that melatonin is widely distributed in the plant kingdom, acting in many morphological and physiological processes (Hattori et al., 1995; Chen et al., 2003; Iriti, 2009; Hardeland et al., 2012; Zuo et al., 2014; Vigentini et al., 2015). Like animals, it shows daily rhythmic fluctuations in its production and function in plants as a cellular protectant against free radicals and oxidation (Mercolini et al., 2012; Zhao et al., 2013; Liang et al., 2015). Exogenously applied or endogenously induced melatonin enhances plant resistance to drought (Wang et al., 2013b; Zuo et al., 2014), salt (Li et al., 2012), cold (Kang et al., 2010; Bajwa et al., 2014), and oxidative stresses (Hardeland et al., 1993; Park et al., 2013), and also delays leaf senescence (Byeon et al., 2012; Wang et al., 2013a; Liang et al., 2015; Shi et al., 2015a). Beyond that, the changes in melatonin levels during seed germination, as well as flower and fruit development indicate specific, mediatory roles in plant growth and development (Zhang et al., 2013; Byeon and Back, 2014b; Wei et al., 2015). Strikingly, it has been suggested that melatonin functions in root development including primary root growth and lateral root formation (Park and Back, 2012; Zhang et al., 2013, 2014).

Roots are essential to plants for many physiological functions, such as anchoring and mechanical support, water and nutrient uptake, and in some cases carbohydrate storage (Hochholdinger et al., 2004; De Smet et al., 2012; Gao et al., 2014). They also serve as the primary interface to sense and respond to unfavorable soil environments, enabling plants to overcome stress challenges (Raven and Edwards, 2001; Malamy, 2005). Thus, a well-developed root system is extremely critical for maintaining vegetable growth, improving crop yield, and optimizing agricultural land use. The architecture of root systems is controlled by a number of endogenous factors and also influenced by several external factors, especially environmental stimuli, such as the availability of water and nutrients (Osmont et al., 2007; Petricka et al., 2012; Gao et al., 2014).

Auxin is characterized as a “root-forming phytohormone” that plays a central role in shaping the root architecture (Xie et al., 2000; Overvoorde et al., 2010). The predominant form of auxin in plants is indole-3-acetic acid (IAA) (Wang et al., 2015), and genetic and biochemical studies of biosynthetic and signaling genes clearly demonstrated that IAA is a key component of endogenous factors that regulate root growth and development (Overvoorde et al., 2010). In plants, melatonin has many similarities with IAA, since both are indole-compounds and share a common biosynthetic route through the compound tryptamine in the tryptophan (Trp)-dependent IAA biosynthetic pathway (Murch et al., 2000; Tan et al., 2016). Arnao and Hernandez-Ruiz thus proposed that melatonin may have auxin-like functions in the regulation of plant growth and development (Arnao and Hernandez-Ruiz, 2006). This hypothesis about the properties melatonin has been demonstrated by several recent studies (Hernandez-Ruiz et al., 2004, 2005; Arnao and Hernandez-Ruiz, 2007). For example, similar to IAA, melatonin can promote the growth of shoots in canary grass, wheat, oat (Hernandez-Ruiz et al., 2005), soybean (Wei et al., 2015), and rice (Liang et al., 2015), while it has a distinctly inhibitory growth effect on pre-existing roots (Hernandez-Ruiz et al., 2005). Furthermore, melatonin promotes lupine hypocotyl growth and regeneration in a dose-dependent manner (Hernandez-Ruiz et al., 2004). Melatonin thus acts as an important regulator of root architecture in the same manner as an auxin.

Rice (*Oryza sativa*) has a typical fibrous root system comprised of embryonic roots, crown roots, and lateral roots (Gao et al., 2014). Root architecture is one of the primary morphological traits to respond to inconsistent or unusual developmental cues or unfavorable environmental conditions, and is closely correlated with rice yield (Gao et al., 2014). Melatonin was recently reported to be a crucial regulator of root developmental processes in rice (Park and Back, 2012). However, to date, mechanistic details of how this molecule regulates root growth remain largely undetermined. In this study, we provide insight into the molecular events associated with the action of melatonin in mediating root growth and development in rice. Genome-wide expression profiling analysis clearly demonstrated that melatonin controls root architecture by modulating auxin response to promote lateral root development and inhibit embryonic root growth. Elucidation of the molecular mechanisms mediated by melatonin will deepen our understanding of the role of this molecule in root growth and development, and further facilitate the application of control over root architecture control for agricultural plants.

## Materials and methods

### Plant material and treatment of melatonin

Rice seedlings of Dongjin (*Oryza sativa* ssp. *Japonica*) were germinated and grown in a growth chamber with a 12-h-light (30°C)/12-h-dark (28°C) photoperiod, with approximately 200 μmol photons/m^2^/sphoton densities and 70% humidity. We previously reported that melatonin delays rice leaf senescence and cell death, and enhances salt stress tolerance by directly or indirectly counteracting the cellular accumulation of H_2_O_2_ (Liang et al., 2015). We also found that a low concentration of melatonin (<20 μM) increased shoot growth, while a high concentration (>20 μM) can mitigate its growth-promoting effect or even have an inhibitory effect (Liang et al., 2015). Therefore, different concentrations of melatonin (0, 10, 20, and 50 μmol/L) were added to the hydroponic cultures when seminal roots of seedlings had reached 2–3 cm. Seminal root length and crown root number were recorded every day. Lateral root length was represented by mean length of the longest three lateral roots about 2 cm from the root tip. Lateral root number was represented by the number of all lateral roots in 1–2 cm region from the root tip. These two parameters were recorded 5-days after treatment. All data were recorded using 30 seedlings. For all experiments, the overall data were statistically analyzed in the SPSS 20 program (SPSS Inc., Chicago, IL, USA). LSD and Tukey's *post-hoc* test were used for testing the differences in growth and root developmental responses during different melatonin treatments.

### RNA sequencing and data analysis

RNAs extracted from roots of 2-week-old seedlings treated with water (M0), 10 μmol melatonin (M10), or 20 μmol melatonin (M20) were used for RNA sequencing. For direct comparison, three libraries, M0, M10, and M20, with different melatonin concentrations for each treatment, were prepared in the same manner and run side by side by BGI Company (Shenzhen, China) on Illumina Hiseq 2000 platform. Differentially expressed genes (DEGs) were analyzed by the Cufflinks software with the fragments per kilo-base per million reads (FPKM) measurement: FPKM = 109 C/NL, where “C” is the number of mapped fragments for a certain gene, “N” is the total reads mapped to the entire genome, and “L” is exon length of a certain gene.

To perform clustering analysis, the expression abundance of each gene was calculated after a pseudo-count of 1 was added to the raw FPKM value for each gene, with the application of log_2_ transformation and *z*-score normalization by the following formula:

(1) $$V^{\prime }=\frac{\log_{2}(V+1)}{\sum_{i=1}^{n}\log_{2}(v_{i}+1)}$$

where *V* = (*v*_1_, …, *v*_*n*_) is the original raw FPKM abundance estimation of the transcript and *V*′ is the new normalized density vector. The Silhouette function was used to select an optimized number of clusters. As a result, eight clusters were obtained using *K*-means clustering analysis (Ranzani et al., 2015). GO enrichment analysis was performed using in-house perl scripts with known gene function annotations downloaded from PlantGSEA and the BinGO plugin provided in Cytoscape (Yi et al., 2013). To find known *cis*-regulatory motifs within up-regulated and down-regulated genes, the promoter region (1000 bp upstream from the transcription start site) of each gene and the entire genome was scanned with known motifs extracted from both AGRIS and PLACE (Higo et al., 1999; Palaniswamy et al., 2006). The significance level was calculated using Fisher's exact test based on the hypergeometric distribution hypothesis. Transcription factor families were downloaded from the Plant Transcription Factor Database (PlantTFDB; Jin et al., 2014).

### RNA extraction, cDNA preparation, and gene expression analysis

Twenty plants were collected for RNA isolation. Total RNA was extracted using the TRIzol reagent (Invitrogen, Carlsbad, CA, USA). RNA was reverse transcribed using the ReverTra Ace qPCR RT Master mix with gDNA Remove Kit (Toyobo, Osaka, Japan). For quantitative real-time PCR (qRT-PCR), SYBR Green I was added to the reaction mix and run on a Chromo4 real-time PCR detection system according to the manufacturer's instructions (CFX96; Bio-Rad, California, USA). The data were analyzed with Opticon monitor software (Bio-Rad). Rice *ACTIN1* was used as an internal control. The primers used for qRT-PCR are listed in Supplementary Table 8. Values are mean ± *SD*. of three biological repeats. Student's *t*-test was used for statistical analysis. Asterisks indicate statistically significant differences compared with wild type (^*^P ≤ 0.05; ^**^P ≤ 0.01).

### Root gravitropism assay

To assess the root gravitropic response, wild-type seedlings were grown vertically until the length of roots reached 3–4 cm and arranged parallel on filter paper infiltrated with 0, 10, 20, and 50 melatonin, respectively. Then seedlings were gravistimulated with 90° rotation. After 24 h, the root curvature of seedlings with different treatments was quantified and compared. This experiment was performed with a population of more than 30 seedlings per treatment.

## Results

### Melatonin participated in shaping rice root architecture

**Figure 1:**
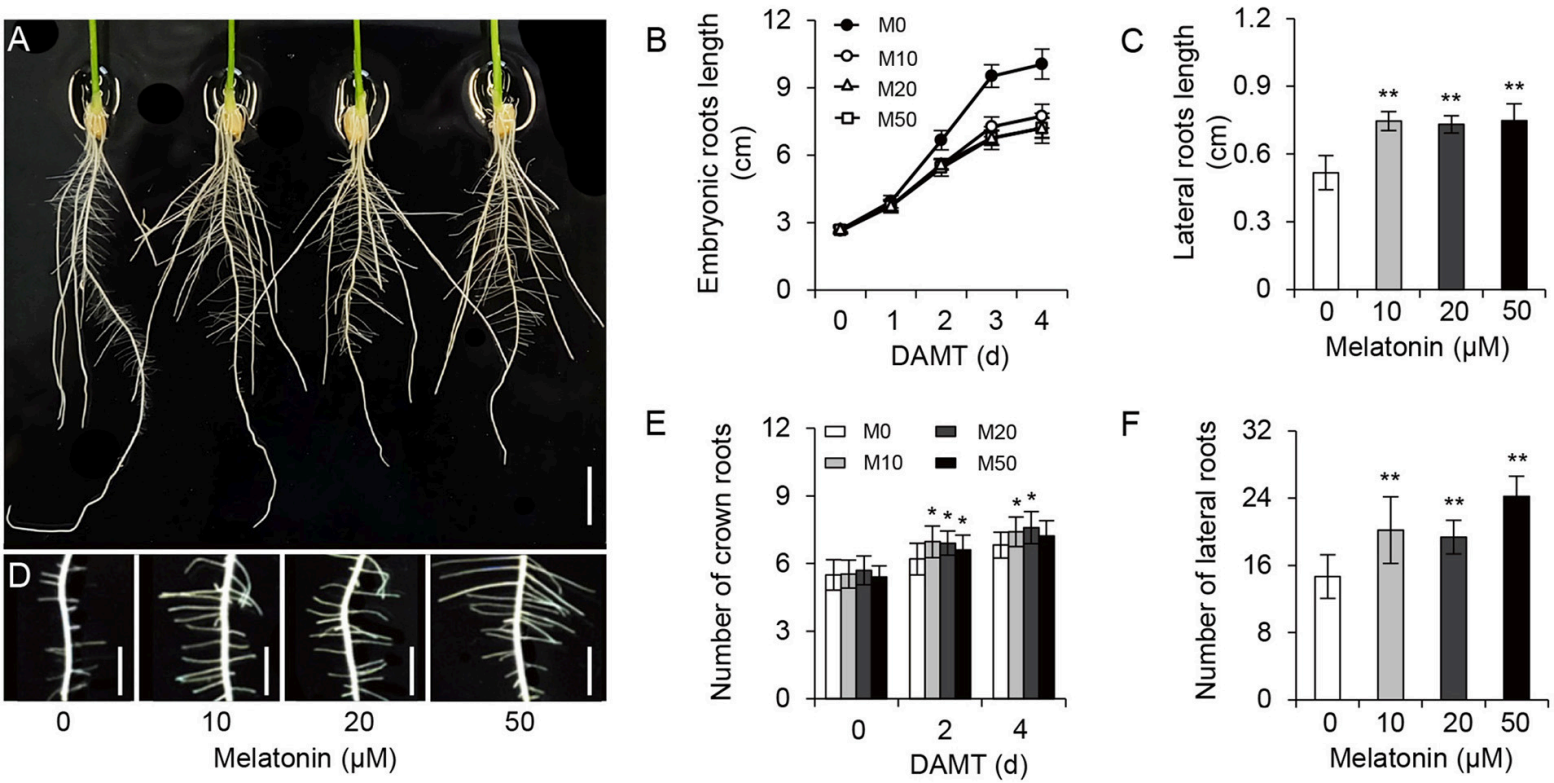
**Melatonin effects on root growth and development in rice. (A)** Phenotypes of rice root architecture with 4-days melatonin treatment. Scale bar = 1 cm. **(B)** Embryonic root length of control and plants cultivated under different concentrations of melatonin. **(C)** Lateral root length of control and plants treated with 10, 20, and 50 μM melatonin for 5 days. **(D)** Phenotypes of lateral roots after 5-days melatonin treatment. Scale bar = 3.6 mm. **(E,F)** number of crown root **(E)** and lateral root **(F)** for 5-days of 0, 10, 20, and 50 μM melatonin treatment. DAMT, days after melatonin treatment. Values are mean ± *SD* of 20 measurements. M0, samples treated with water. M10, samples treated with 10 μM melatonin. M20, samples treated with 20 μM melatonin. M50, samples treated with 50 μM melatonin. ^*^P ≤ 0.05, ^**^P ≤ 0.01. Student *t*-test was used to generate *P*-value.

To examine the effect of melatonin on rice root architecture, we treated rice seedlings with a concentration gradient of melatonin. Visual observation and statistical analysis showed that the embryonic root lengths were significantly decreased with both low and high concentrations of melatonin application, compared with the control treatment (M0; Figures 1A,B and Supplementary Figure 1). Likewise, the average length of crown roots also clearly inhibited with treatment (Figure 1A). In contrast to the decreased length of embryonic and crown roots, the plants treated with melatonin had longer lateral roots on average than the M0 control at 5-days after treatment (Figures 1C,D). Moreover, M10-, M20-, and M50-treated plants showed more crown roots (Figures 1A,E) and lateral roots than the M0 control (Figures 1D,F). These data strongly suggest that melatonin plays an important role in root architecture.

### RNA-seq analysis of a melatonin-treated rice transcriptome

**Figure 2:**
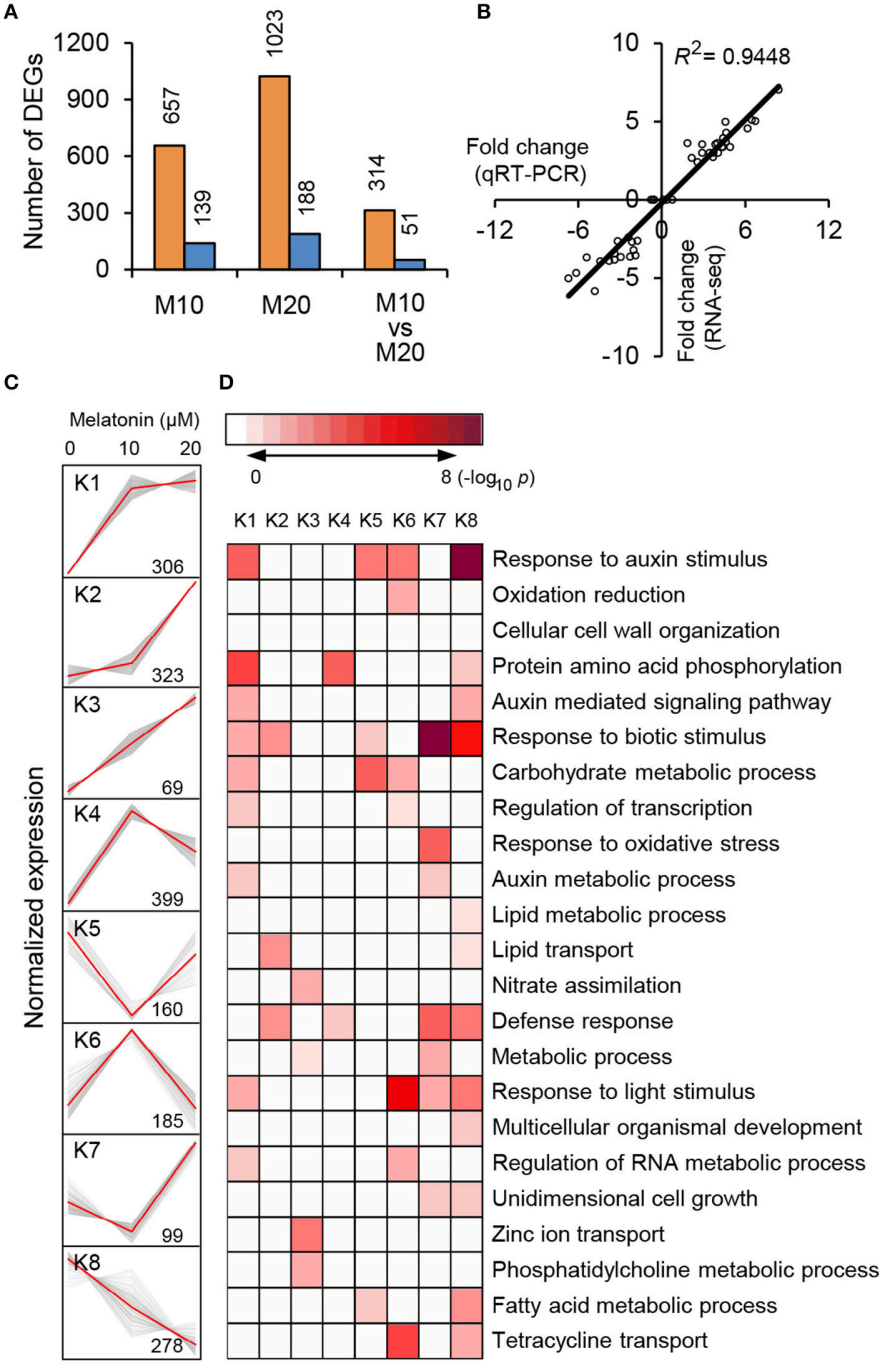
**RNA-seq analysis of melatonin-treated transcriptome. (A)** Overlapping DEG numbers between M0/M10 andM0/M20. **(B)** Correlation of RNA-seq (*y*-axis) and qRT-PCR date (*x*-axis). The correlation assay was carried out for 36 DEGs with log_2_ ratios >1.0 or <1.0. **(C)** *K*-mean clustering showing the expression pattern of the DEGs of M10 and M20 transcriptome in rice roots. Eight clusters were identified with 10 and 20 μM melatonin treatment from 2007 differentially expressed genes. **(D)** Functional category enrichment among the eight major clusters.

To explore the morphological effects of melatonin on root growth and development in rice, we performed RNA-seq using M0-, M10-, and M20-treated roots (Supplementary Table 1). Compared with transcripts of non-treated samples (M0), 796 differentially expressed genes (DEGs), with 2-fold or higher changes, were identified in the M10-treated samples, while 1211 DEGs were identified in M20-treated roots (Figure 2A and Supplementary Table 2). In both M10 and M20 plants, up-regulated genes outnumbered down-regulated genes approximately 4.7- and 5.4-fold in each sample, respectively (Figure 2A). Notably, 314 of the up-regulated and 51 of the down-regulated DEGs overlapped between M10- and M20-treated samples (Figure 2A). Furthermore, Quantitative real-time (qRT)-PCR was performed to validate these DEGs. Forty-four genes with different changes of expression levels under 10 or 20 μmol/L melatonin treatments were randomly selected for qRT-PCR analysis (Supplementary Table 2). As shown in Figure 2B, the regression slope for RNA-seq vs. qRT-PCR is close to 1, suggesting a high positive correlation between RNA-seq data and qRT-PCR data, thus demonstrating the credibility of the RNA-seq data.

To test the efficacy of melatonin we assign genes to functional categories and grouped the DEGs from M10 and M20 using the *K*-Means clustering algorithm. The 1819 (90.6%) DEGs were mainly clustered into eight groups (K1–K8; Figure 2C and Supplementary Table 3). Most of the bins exhibited enrichment for specific clusters of expressed genes. Cluster 1, 2, 3, and 4 showed patterns of up-regulation, while cluster 5 and 8 exhibited down-regulated gene expression profiles. In cluster 6, genes with induced expression in M10 samples are inhibited in M20 samples, whereas genes in cluster 7 were down-regulated in M0 but up-regulated in M20. Genes in cluster K1 and K8 include those for response to stimuli, hormone-mediated signaling pathways, protein amino acid phosphorylation, defense response, and metabolic processes. Genes in clusters K2, K3, and K6 include those encoding nutrition absorption and transport. Significantly, genes encoding enzymes for response to auxin stimulus, auxin-mediated signaling pathway, and auxin metabolic processes are greatly enriched in cluster K1, 6, and 8, K1 and 8, K1 and 7, respectively. These observations imply that melatonin regulates the expression of these genes to govern root development in rice.

**Figure 3:**
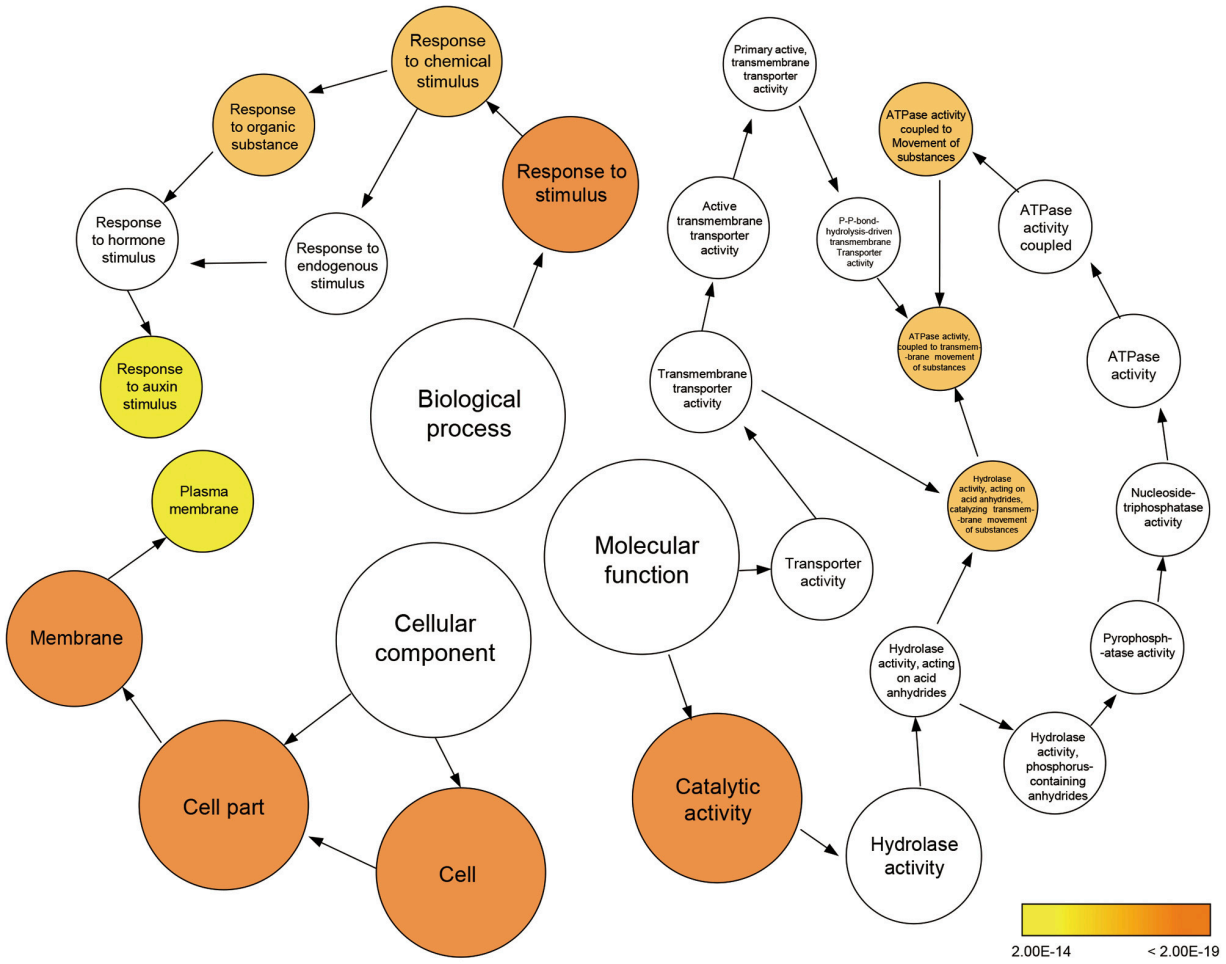
**Enriched gene ontology (GO) terms for the up-regulated genes in the M10 vs. M20**. Node size reflects the number of genes that belong to the category. Colored nodes represent GO terms that are significantly over-represented (*P* < 0.05), and the color scale indicated increasingly higher statistical significance.

To further clarify the plant gene expression responses to melatonin, we used BinGO to construct gene ontology (GO) term networks for DEGs of M10 and M20 plants, as well as overlapping DEGs between M10 and M20. As expected, GO terms correlated with response to auxin stimulus were highlighted in the biological process category among the three groups. Genes encoding ATPase activity were clearly over-represented in the molecular function category. Eighteen genes annotated as response to auxin stimuli and auxin signaling pathway, were dominant as the main categories of biological process (Figure 3 and Supplementary Table 4). The array of genes associated with auxin-related processes suggests that melatonin might regulate root architecture by affecting auxin signaling in rice.

### Expression pattern of transcription factors

**Figure 4:**
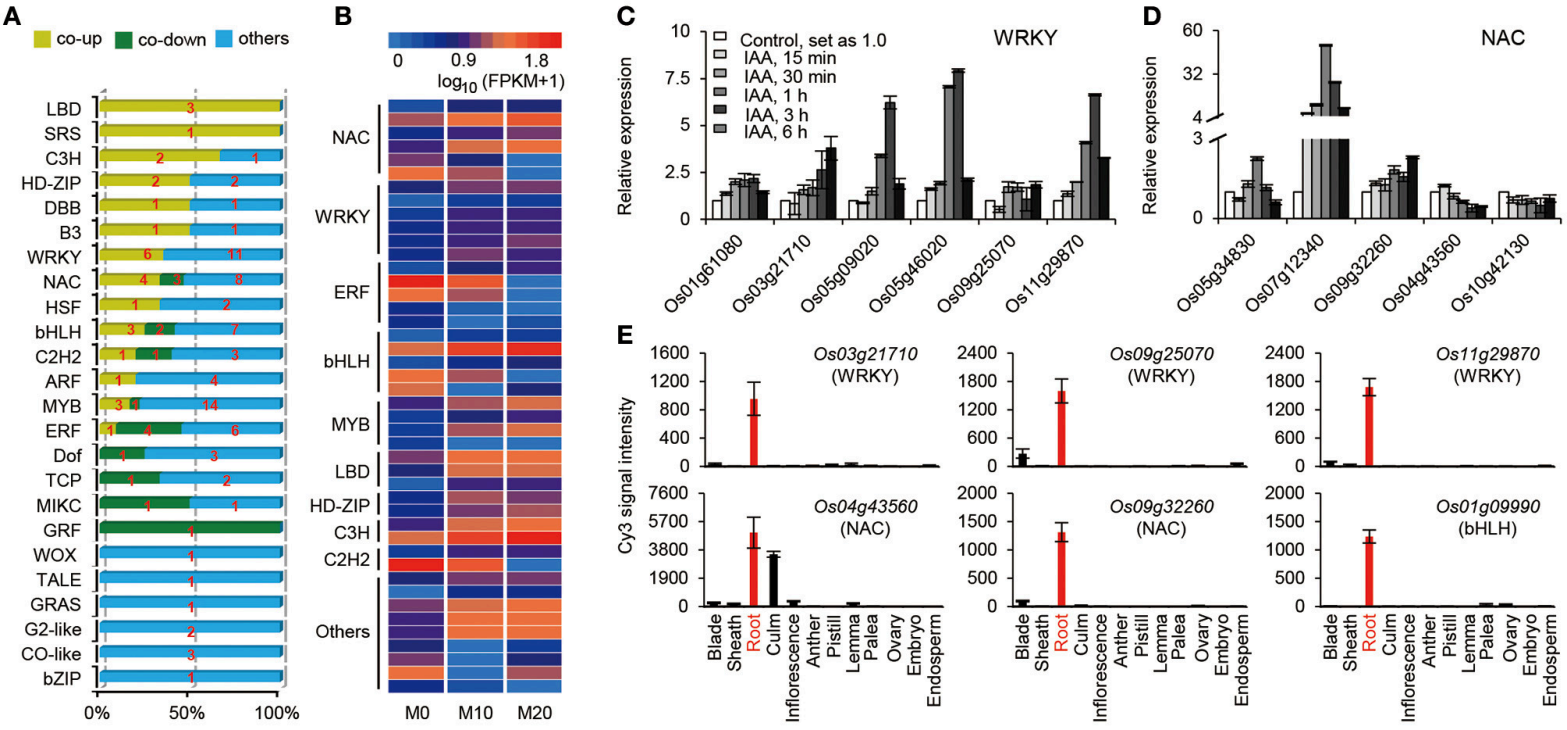
**The expression profiles of transcription factors (TFs) regulated by melatonin. (A)** Distribution of the transcription factor families among M0, M10, and M20. **(B)** The heat map of co-up and co-down expression of TFs in M10 and M20. Detailed annotation information of genes can be found in Supplementary Table 4. **(C,D)** Expression of the WRKY **(C)** and NAC **(D)** family genes in co-up and down transcription factors. The data were extracted from RiceXPro (http://ricexpro.dna.affrc.go.jp/). **(E)** Expression of root specific genes in co-up and down TFs. The data were extracted from RiceXPro (http://ricexpro.dna.affrc.go.jp/).

Transcriptional activators and repressors have vital roles in regulating gene expression surrounding both the melatonin-mediated biological processes and shaping the plant root architecture (Gao et al., 2014; Liang et al., 2015). A total of 120 TFs belonging to 24 families, were up- or down-regulated in the M10 and M20 samples compared with M0 control (Figure 4A and Supplementary Table 5). The expression of roughly 25% (30) of TFs were significantly activated in both M10 and M20, including six WRKY, four NAC, three MYB, three bHLH, three LBD, two C3H, two HD-ZIP, one SRS, one B3, one HSF, one DBB, one C2H2, one ARF, and 1 ERF, while 14 (11.7%) TFs were inhibited (Figures 4A,B). Intriguingly, the expression of many auxin-induced TFs (http://ricexpro.dna.affrc.go.jp/), such as *Os01g61080* (WRKY), *Os03g21710* (WRKY), *Os05g09020* (WRKY), *Os05g46020* (WRKY), *Os09g25070* (WRKY), *Os11g29870* (WRKY), *Os05g34830* (NAC), *Os07g112340* (NAC), *Os09g32260* (NAC), and *Os01g09990* (bHLH), were significantly upregulated in the both M10 and M20 samples (Figures 4B,C), whereas the expression of several auxin-inhibited TFs, including *Os04g43560* (NAC), *Os10g42130* (NAC), *Os02g43940* (ERF), *Os02g51280* (TCP), *Os04g23910* (MIKC), *Os02g53690* (GRF), and *Os06g17410* (DOF), were down–regulated in both samples (Figures 4B,C). These data further confirmed the relationship between melatonin signaling and auxin response in rice. Notably, among 44 co-up- or co-down- regulated TFs, 21 genes, such as *Os03g21710* (WRKY), *Os09g25070* (WRKY), *Os11g29870* (WRKY), *Os04g43560* (NAC), *Os09g32260* (NAC), *Os01g09990* (bHLH), *Os04g23910* (MIKC), and *Os02g51280* (TCP), were specifically or primarily expressed in roots (Figures 4B–D and Supplementary Table 5), implying that these TFs may be potential key regulators of melatonin signaling pathway. Taken together, these results provide strongly evidence that melatonin acts as an important regulator of root development in a partially auxin-dependent auxin manner in rice.

### Identification of melatonin-associated motifs in co-expressed genes

**Figure 5:**
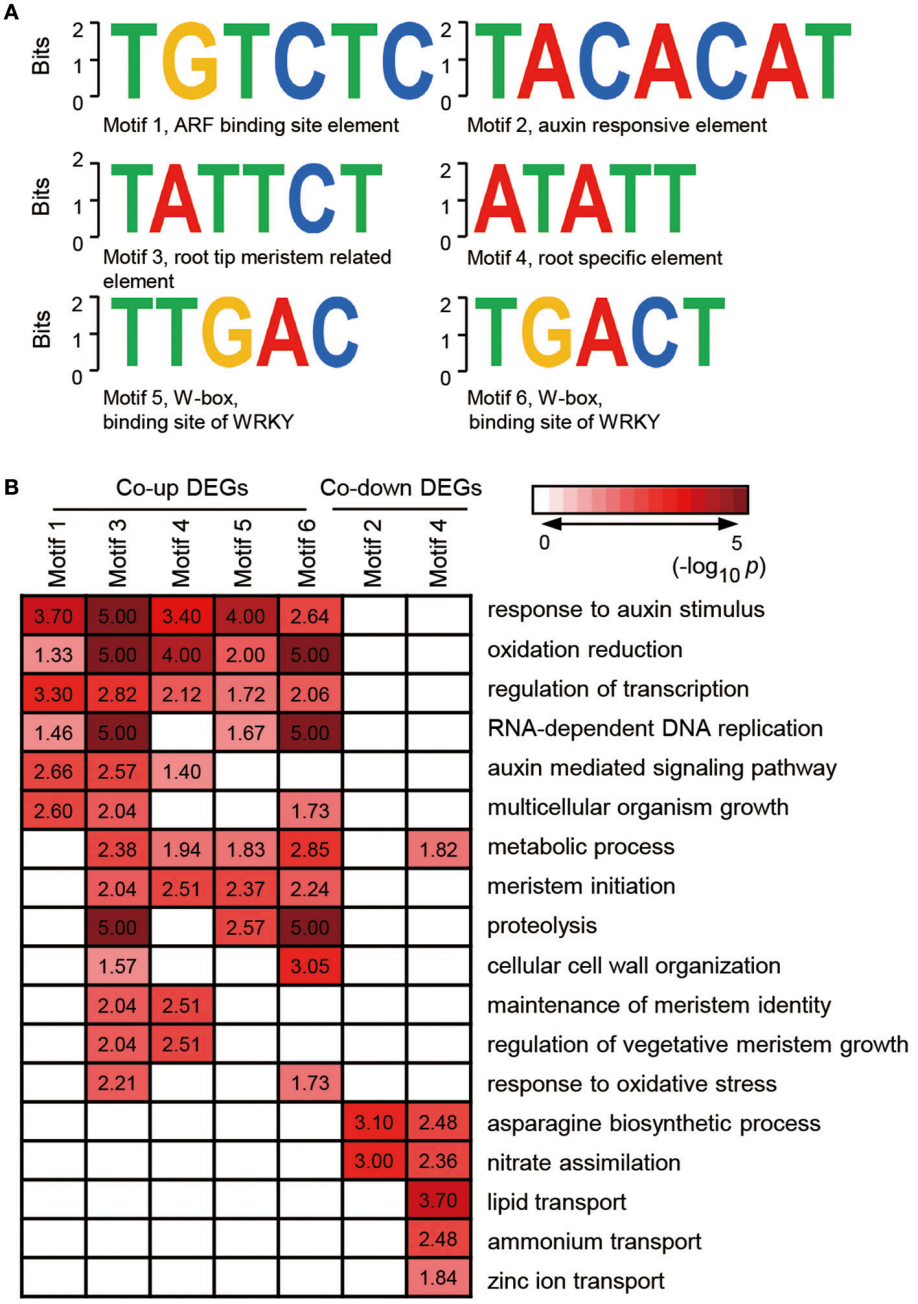
**Discovered candidate motifs from co-up and co-down DEGs and enriched analysis of genes with the motifs binding sites. (A)** Candidate *cis*-elements identified by ELEMENT from co-up and co-down DEGs. **(B)** GO annotation enrichment analysis for the genes containing motif1 to motif6. DEGs, differentially expressed genes.

Given that genes with similar expression profile may contain a conserved *cis*-element in their promoters, we screened the 1000 bp sequences upstream of the transcriptional start site of co-expressed genes or co-inhibited genesby AGRIS and PLACE. We then submitted the candidate motifs to the motif searching program to identify statistically over-represented regulatory motifs. Fifty-eight and twenty-one candidate *cis*-elements were identified in co-up and co-down DEGs, respectively (Supplementary Table 6). Significantly, three known conserved motifs, including an ARF binding site motif (motif1, TGTCTC), a root tip meristem-related element (motif3, TATTCT), and a root-specific element (RSE, motif4, ATATT), were found to be enriched in promoters of 79, 95, and 266 co-expressed DEGs (Figure 5A and Supplementary Table 7). Similarly, a conserved sequence, TACACAT (motif2), required for auxin responsiveness, and the RSE motif, were identified in 17.6% (9) and 90.2% (46) of co-inhibited DEGs, respectively (Figure 5A). These data further demonstrate that melatonin functions in roots architecture in an auxin-related manner. In addition, a conserved W-box (motif5, TTGAC and motif6, TGACT), the core binding site of WRKY TFs, was enriched in co-up DEGs (Figure 5A and Supplementary Table 7), which correspond well with the GO results showing that WRKY families were the most highly enriched of all TFs in the co-up TFs (Figures 4B,C).

Further analysis of the seven clusters of genes containing motif 1–6 showed that the co-up-regulated genes harboring promoter motifs 1, 3, 4, 5, or 6 were commonly enriched among GO terms such as response to auxin stimulus, oxidation reduction, and regulation of transcription, while the co-down genes with motifs 2 and 4 are enriched in GO term pathways related to the asparagine biosynthetic process and nitrate assimilation (Figure 5B). Genes encoding enzymes for the auxin-mediated signaling pathway, metabolic process, meristem initiation, maintenance of meristem identity, and regulation of vegetative meristem growth are greatly enriched in motif3 and motif4 clusters, which contain *cis*-elements related to root development (Figure 5B). Several GO terms associated with auxin response and root development showed significant enrichment in genes carrying motif5 and motif6, suggesting that the WRKY TFs may also participate in melatonin-mediated growth response in rice.

### Melatonin affects auxin-regulated processes in rice

**Figure 6:**
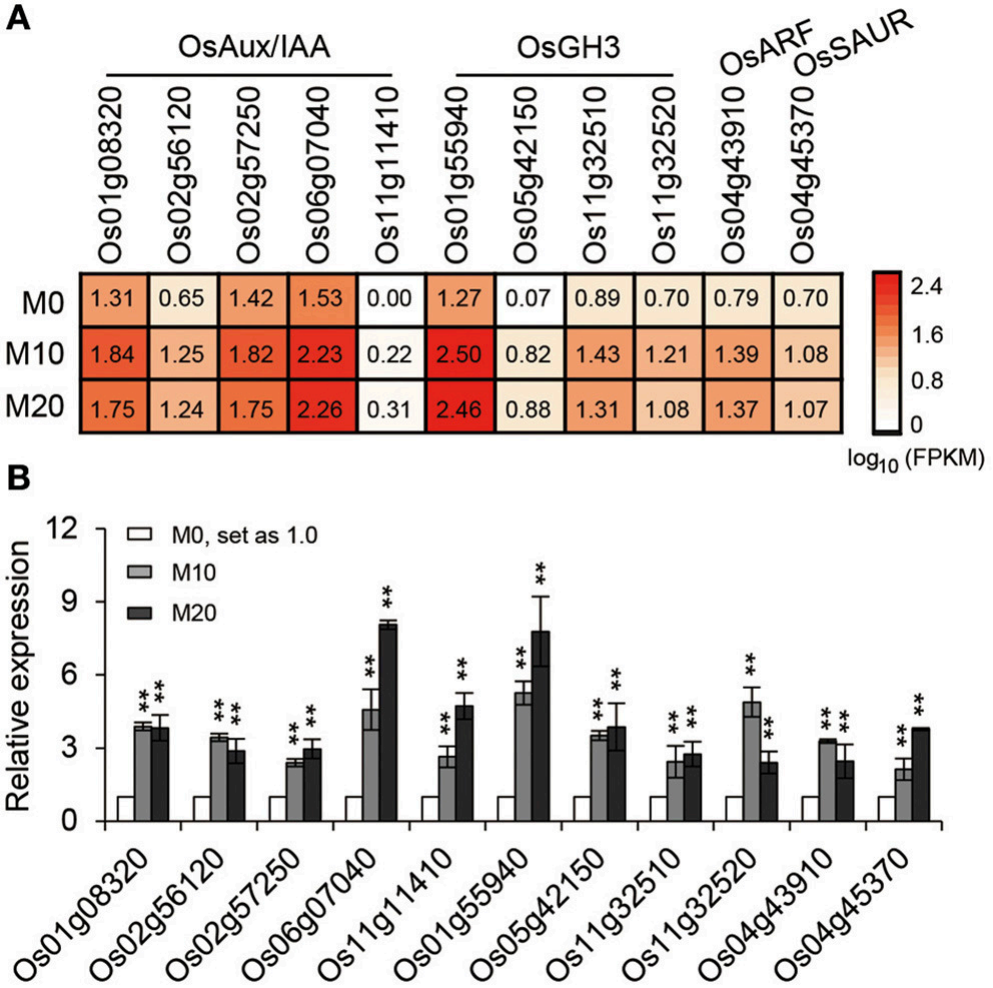
**The relative expression pattern of auxin signaling pathway related genes. (A)** The relative expression profile of co-up regulated auxin signaling genes in rice root in M0, M10, and M20, including five *OsAux/IAA*, four *OsGH3*, one *OsARF*, and one *OsSAUR* genes. **(B)** Relative transcript levels of genes corresponding to **(A)** by qRT-PCR. M0, samples treated with water. M10, samples treated with 10 μM melatonin. M20, samples treated with 20 μM melatonin. Transcript levels are expressed relative to that of rice *ACTIN1* in each sample, and values are mean *SD* (*n* = 3). ^**^P ≤ 0.01. Student *t*-test was used to generate *P*-value.

The transcriptional response data brought to light the possibility that melatonin regulates root architecture in an auxin dependent interaction. To further explore this hypothesis, we retrieved genes involved in the auxin biosynthesis and signaling pathways in co-transcribed DEGs, based on previous reports, to analyze their expression patterns (Supplementary Table 2). Strikingly, we found that the expression of several classes of auxin-related genes, including five *Aux/IAA* members, four *OsGH3* members, one *ARF*, and one *SAUR* gene involved in the auxin signaling pathway were all notably up-regulated both in M10 and M20, compared with M0 control (Figure 6A). The activated transcripts for these genes were further confirmed by qRT-PCR (Figure 6B). However, no remarkable change was observed in the genes involved in IAA biosynthesis and metabolism pathways. This result confirms that melatonin regulates root development in rice, probably acting in a manner dependent on auxin signaling.

### Melatonin regulates root architecture by modulating auxin signaling

**Figure 7:**
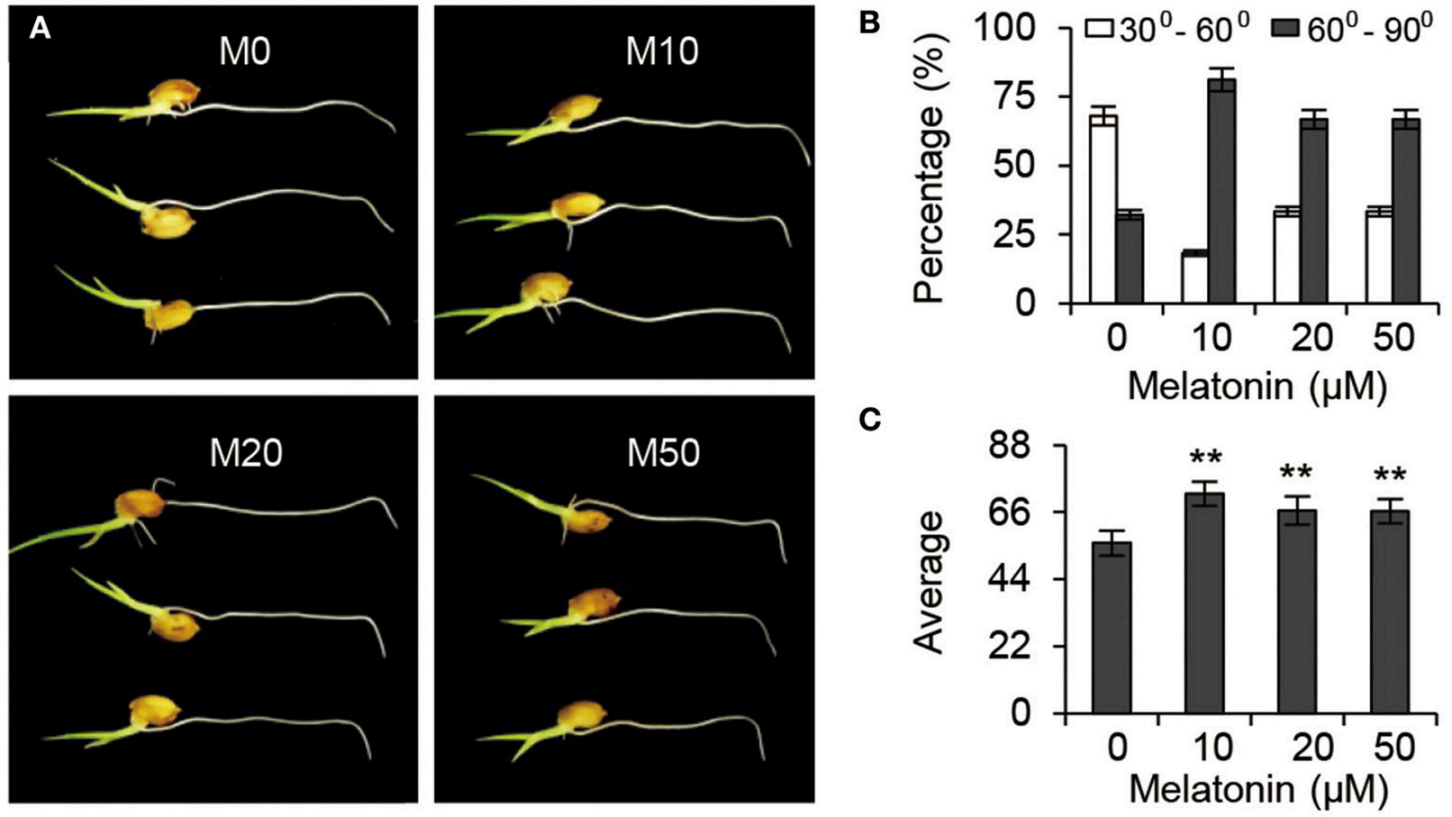
**Auxin responses under melatonin treatment. (A)** Gravisensitivity of seedling roots under melatonin treatment. M0, M10, M20, and M50 seedlings were grown vertically for 3-days and then rotated 90°. M0, samples treated with water. M10, samples treated with 10 μM melatonin. M20, samples treated with 20 μM melatonin. M50, samples treated with 50 μM melatonin. **(B)** Statistics for the root tip angles in **(A)** at 24 h after reorientation. **(C)** Average of the root tip angles in corresponding to **(A)**. ^**^P ≤ 0.01. Student *t*-test was used to generate *P*-value.

Gravitropic responses have been widely used as a reporter for auxin homeostasis or signal transduction in plants. We examined the root gravitropic response in M10 and M20 plants by measuring the curvature after gravistimulation at 90° to the vertical for 24 h. About 67.9% of M0 roots had root tip angles of 30° to 60°, while 72.8, 66.7, and 66.7% of M10, M20, and M50 roots, respectively, were observed to have roots tip angles of 61–90% (Figures 7A,B). The average root tip angles of the M10 were 55.9°, whereas M10, M20, and M50 roots had respective average angles 71.9°, 66.5°, and 66.4° (Figure 7C). The difference in root tip angles between treatments and control clearly demonstrated that melatonin mediates root growth and development processes through effects on the auxin signaling pathway.

## Discussion

Melatonin is a ubiquitous and physiological compound and is proposed to be an important regulator in controlling root development (Arnao and Hernandez-Ruiz, 2007; Park and Back, 2012; Zhang et al., 2013). However, both the mechanistic evidence of melatonin's regulatory role in root architecture and the molecular interactions driving melatonin-mediated root development remain largely unknown. In our study, embryonic root and crown root elongation were inhibited significantly by exogenous melatonin treatment, while the number and length of lateral roots were distinctly increased both in M10 and M20 plants, compared with the M0 control (Figure 1). The role of melatonin in orchestrating rice root architecture was highly similar with the most well-characterized auxin-associated phenotypes, such as increased length of epidermal-derived root hairs, inhibited growth of pre-existing primary roots, and increased number of lateral roots (Overvoorde et al., 2010). As demonstrated by the enhanced gravitropic response in M10, M20, and M50 (Figure 7), melatonin may have a function similar to auxin in root developmental regulation, and consistent with auxin-related processes.

Auxins are known to be critical phytohormones involved in regulating root development. A number of auxin-related mutants, such as *Arabidopsis crownless root1 (crl1)/adventitious rootless1 (arl1)* (Inukai et al., 2005; Liu et al., 2005), *crl4/gnom1*(Liu et al., 2009), and *root enhancer1(ren1-D)* (Gao et al., 2014), show abnormalities in root growth and development. Several lines of evidence in our RNA-seq data support the idea melatonin shared function with auxin. First, a large proportion of DEGs were determined to be involved in the response to auxin stimulus and the auxin mediated signaling pathway during melatonin treatment (Figures 2, 3). Second, based on our enrichment analysis, several classes of auxin responsive genes, including five *OsAux/IAA*, four *OsGH3*, one *OsARF*, and one *OsSAUR*, were significantly up-regulated in both M10 and M20 compared with M0 control (Figure 6 and Supplementary Table 2). The ARF, IAA/Aux, GH3, SAUR genes are the most important auxin signaling and response gene families in plants. The regulation of post-embryonic root growth and lateral root formation is closely controlled by auxin signaling. For example, gain-of-function mutations *iaa1/axr5* (Yang et al., 2004), *iaa3/shy2* (Tian and Reed, 1999), *iaa14/slr* (Fukaki et al., 2002), *iaa18/crane* (Uehara et al., 2008), *iaa19/msg2* (Tatematsu et al., 2004), and *iaa28* (Rogg et al., 2001), exhibited an obviously altered capacity to form lateral roots. A recent discovery showed that melatonin inhibits the transcripts of an AUX/IAA gene, *IAA17*, to delay natural leaf senescence in *Arabidopsis* (Shi et al., 2015a). Several OsAux/IAA genes, including *Os01g08320* (*OsIAA1*), *Os02g56120* (*OsIAA9*), *Os02g57250* (*OsIAA10*), *Os06g07040* (*OsIAA20*), and *Os011g11410* (*OsIAA27*), were significantly upregulated in our analysis, demonstrating that activity in plant development may be through an auxin signaling pathway. However, some reports suggest that melatonin does not regulate AXR3/IAA17 nor activate auxin-inducible gene expression in root development in *Arabidopsis* (Pelagio-Flores et al., 2012). The differences may be caused by the differential expression profiles of OsAux/IAA genes. In plants, the Aux/IAA members have tissue-specific expression patterns, and distinct functions in auxin-mediated growth and development. Alternatively, this discrepancy also might be due to different mechanisms for melatonin regulation of root development between species. Comparative transcriptome analysis revealed that there are the differences in gene expression and hormone signaling pathways during root development in different plant species. Third, the expression of many auxin-related TFs (http://ricexpro.dna.affrc.go.jp), including WRKY, NAC, MYB, bHLH, HD-ZIP, and ERF, exhibited consistently up- and down-regulated expression profiles under both M10 and M20 treatment, respectively (Figure 4). Fourth, two conserved auxin-related *cis*-elements, an ARF binding site motif (TGTCTC) and an auxin-response element (TACACAT), were identified in 25.2% (27) co-up and 17.6% (9) co-down DEGs, respectively (Figure 5). Based on these results, we can reasonably speculate that melatonin-controlled root growth and developmental regulation is closely associated with the activation of auxin response pathway in rice.

In addition, we observed enrichment of several up-regulated genes containing “root tip meristems” and “root-specific element (RSE)” promoter motifs associated with GO terms categories meristem initiation, oxidation reduction, RNA-dependent DNA replication, and proteolysis. Significant enrichment was also observed in down-regulated genes having the “RSE” *cis*-element annotated for nitrate assimilation, lipid and zinc ion transport, and the asparagine biosynthetic process. These results further supported our hypothesis that melatonin is an important mediator for shaping root architecture via modulation of auxin response in rice.

In spite of recent progress in elucidating the biological function of melatonin, understanding of the molecular mechanisms of melatonin-mediated root growth and development is still at a beginning stage. Currently, a limited number of genes for melatonin biosynthesis, degradation, and signaling pathways have been identified using a reverse genetic approach (Kang et al., 2013; Zhao et al., 2013; Byeon and Back, 2014a; Lee et al., 2014; Zuo et al., 2014; Shi et al., 2015a,b). Given that it is difficult to identify melatonin-related genes using a forward genetic approach so far, the identification of critical components controlling melatonin biosynthesis, degradation, and signaling, needs to be accomplished though several different approaches. Our studies suggest that a systems biology approach, especially combining different “omics” methods and CRISPR/Cas9, should accelerate the identification of key genes underlying melatonin biosynthesis, degradation, and signaling. On the other hand, analyses that measure changes in melatonin content and signaling effects in mutants for auxin biosynthesis, degradation, and signaling would also serve as an important means to uncover the relationship between melatonin and auxin.

## Author contributions

Chengzhen Liang and CC conceived and designed the experiments; Chengzhen Liang, AL, WL, and RZ performed laboratory experiments; Chengzhen Liang, AL, HY, and Chengzhi Liang performed data analysis and interpretation; Chengzhen Liang, AL, SG, RZ, and CC wrote the paper.

## Funding

This work was supported by grant from the National Natural Science Foundation of China (No. 31430063 and 31601349), and grant from the Ministry of Agriculture of China (Grant No. 2014ZX0800933B, 2016ZX08009003-003-004).

### Conflict of interest statement

The authors declare that the research was conducted in the absence of any commercial or financial relationships that could be construed as a potential conflict of interest.

## Supplementary material

Supplementary Table 1 **Statistics of clean reads in RNA sequencing**.

Supplementary Table 2 **Differentially expressed genes**.

Supplementary Table 3 **Genes in K-mean clustering of eight clusters**.

Supplementary Table 4 **GO annotation enrichment analysis**.

Supplementary Table 5 **Transcription factors**.

Supplementary Table 6 **Candidate cis-elements discovered from co-up and -down genes**.

Supplementary Table 7 **Candidate Cis-elements discovered by ELEMENT from co-up and co-down genes**.

Supplementary Table 8 **Primers used for qRT-PCR in this study**.

Supplementary Figure 1 **Melatonin effect on rice root grown in greenhouse. (A)** Phenotypes of 2-week-old plants with different concentrations of melatonin. **(B)** Root length corresponding to **(A)**.
