# Supplementary material for: Melatonin Regulates Root Architecture by Modulating Auxin Response in Rice
Source: Front Plant Sci. 2017 Feb 7;8:134. doi: 10.3389/fpls.2017.00134 (PMC5293752; doi:10.3389/fpls.2017.00134)
Supplement: Supplementary Table 1 — Statistics of clean reads in RNA sequencing. [file DataSheet1.PDF]

Supporting information

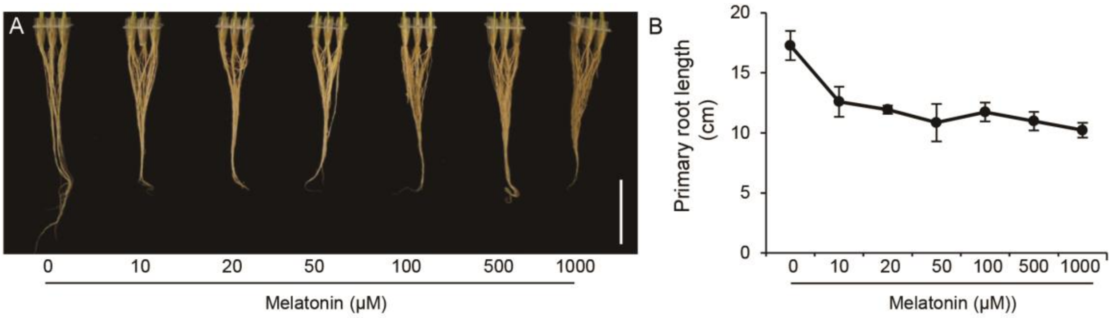

Supplementary Figure 1 Melatonin effect on rice root grown in greenhouse. (A) Phenotypes of 2-week-old plants with different concentrations of melatonin. (B) Root length corresponding to (A).

Supplementary Table 1 Statistics of clean reads in RNA sequencing

Samples	Total reads	Total mapped	Unique mapped	Multiple aligments	Mapped (%)	Unique mapped (%)
M0	16,566,204	12,974,986	12,266,777	708,209	78.32	74.05
M10	16,286,711	12,179,513	11,552,653	626,860	74.78	70.93
M20	15,750,079	12,712,887	12,084,172	628,715	80.72	76.72

M0, samples treated with water. M10, samples treated with 10 μM melatonin. M20, samples treated with 20 μM melatonin.

Supplementary Table 7 Candidate Cis-elements discovered by ELEMENT from co-up and co-down genes

Binding Sites	Annotation	Gene No.	*p*-value
**Co-up**			
TGTCTC	ARF binding site motif	79	5.0E-02
TATTCT	Root tip meristems-related element	95	1.0E-02
ATATT	Root specific element	266	5.0E-04
TTGAC	W-box, binding site of WRKY	192	2.3E-02
TGACT	W-box, binding site of WRKY	179	2.4E-02
**Co-down**			
TACACAT	Auxin responsive element	9	1.5E-02
ATATT	Root specific element	51	3.6E-02

Table S8. Primers used for qRT-PCR in this study.

	Forward Primers	Reverse Primers
ACTIN1	TCCATCTTGGCATCTCTCAG	GTACCCTCATCAGGCATCTG
LOC_Os01g08320	CGCTCCAGGACAAGTTCTTC	CTGGCAAGTTTCCACAAACA
LOC_Os01g55940	GCCGACGATAGAAGATGAGC	ACACGTTGTAGGGGTCGAAG
LOC_Os01g61560	GCCTCTTTTCAGCTCAGGAA	GAGAAGTTCCCTCGGGTCTC
LOC_Os02g56120	TTGCAGGAGGAGGAAGAAGA	TCGTCGTAGGAGGAATGGAG
LOC_Os02g57250	GTCATTGGAAGGAAGGTGGA	GGAACATCTCCAACGAGCAT
LOC_Os04g43910	ACCGAGGAGCAGATCAAGAA	CCAAAATACGGTAGGCTCCA
LOC_Os04g45370	ACTTCAACCACCCCATGTTC	ATCAGCCCATTCTGATGGAA
LOC_Os05g42150	AGCATCGACTCCGACAAGAC	GACCTGAGCTCCCAGAACAG
LOC_Os06g07040	GCAACAAGAGGAGGAAGCTG	TCGTAGGTGACAGCGTATGG
LOC_Os06g49050	TTGCATCAACCTTCTGCTTG	AGGCGAGCTAAACATCTCCA
LOC_Os11g11410	GTACAAGGTGGGCAGGAAGA	TTGCTGATGCAAGGAACAAC
LOC_Os11g32510	ACAAGGTGGCACACGTCAT	TTGTACTGGTTCACCGACGA
LOC_Os11g32520	GGAGTCCTACTTCGGCATCA	CTCACCTGTGAAGGTGGTGA
